# Electrochemical Friedel–Crafts-type amidomethylation of arenes by a novel electrochemical oxidation system using a quasi-divided cell and trialkylammonium tetrafluoroborate

**DOI:** 10.3762/bjoc.18.105

**Published:** 2022-08-18

**Authors:** Hisanori Senboku, Mizuki Hayama, Hidetoshi Matsuno

**Affiliations:** 1 Division of Applied Chemistry, Faculty of Engineering, Hokkaido University, Kita 13 Nishi 8, Kita-ku, Sapporo, Hokkaido 060-8628, Japan; 2 Graduate School of Chemical Sciences and Engineering, Hokkaido University, Kita 13 Nishi 8, Kita-ku, Sapporo, Hokkaido 060-8628, Japan

**Keywords:** electrochemical oxidation, Friedel–Crafts type amidomethylation, *N*-acyliminium ion, quasi-divided cell, trialkylammonium salt

## Abstract

Electrochemical Friedel–Crafts-type amidomethylation was successfully carried out by a novel electrochemical oxidation system using a quasi-divided cell and trialkylammonium tetrafluoroborates, such as iPr_2_NHEtBF_4_. Constant current electrolysis of 1,3,5-trimethoxybenzene or indoles in DMA containing 0.1 M iPr_2_NHEtBF_4_ using an undivided cell equipped with a Pt plate cathode and a Pt wire anode (a quasi-divided cell) resulted in selective formation of *N*-acyliminium ions of DMA at the anode, which reacted with arenes to give the corresponding amidomethylated products in good to high yields.

## Introduction

**Scheme 1:**
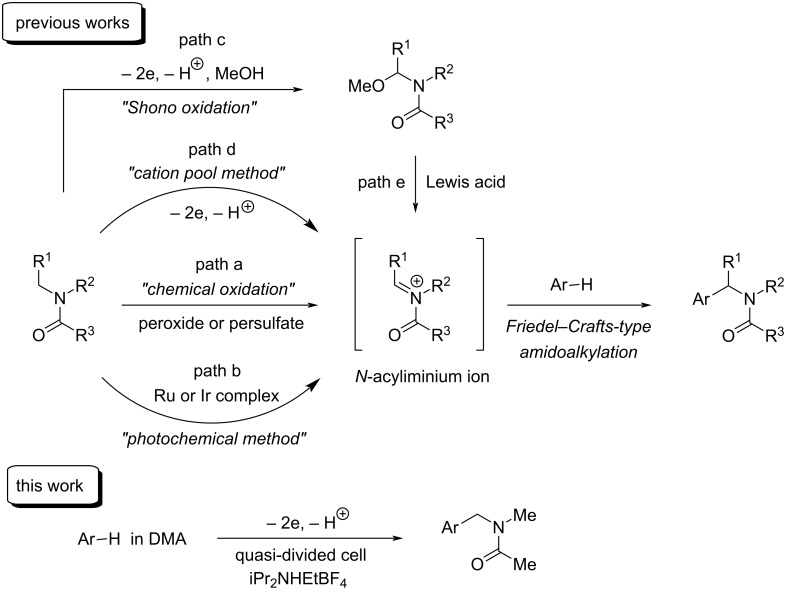
Generation of *N*-acyliminium ion: previous and present works.

Oxidation of amides generates useful intermediates, *N*-acyliminium ions, which have been widely used in organic synthesis [1–4]. For example, Friedel–Crafts-type amidomethylation [5–15] proceeds efficiently by the reaction of *N*-acyliminium ions with electron-rich arenes to give the corresponding amidomethylated products in good yields. Since amides are important intermediates in organic synthesis and sometimes appear in biologically active compounds, pharmaceuticals, agrochemicals and functional molecules, amidomethylation induced by *N*-acyliminium ions is a helpful and valuable protocol for direct introduction of an amide function into organic molecules. Generation of *N*-acyliminium ions in chemical methods has been generally accomplished by the reaction of amides with chemical oxidants, such as peroxides and persulfates at high temperature (path a in Scheme 1) [10–13]. A metal catalyst or a photocatalyst consisting of metals, such as ruthenium or iridium, is also necessary in some cases (path b in Scheme 1) [14–15]. On the other hand, *N*-acyliminium ions can easily be generated by electrochemical oxidation without those reagents. Electrochemical oxidation of amides/carbamates yielding *N*-acyliminium ions is well known as Shono oxidation (path c in Scheme 1) [16] and has also been applied to organic synthesis [17–20]. However, when electrochemical oxidation of amides/carbamates in the presence of nucleophiles, such as electron-rich arenes or silyl enol ethers, is carried out for Friedel–Crafts-type amidomethylation, electrochemical oxidation of electron-rich arenes or silyl enol ethers preferentially takes place at the anode due to their, in general, more positive oxidation potentials than those of amides/carbamates. Therefore, Friedel–Crafts-type amidomethylation by using Shono oxidation is successfully carried out as a two-step process: electrochemical oxidation of amides/carbamates yielding α-methoxylated amides/carbamates (Shono oxidation, path c in Scheme 1) followed by the reaction of the isolated α-methoxylated amides/carbamates with arenes in the presence of a Lewis acid catalyst (path e in Scheme 1) [16]. Although the use of CH_2_Cl_2_ as a solvent and a divided cell with a low temperature (−78 °C) and a relatively high concentration of the supporting electrolyte are necessary, the cation pool method [21] developed by Yoshida and Suga was effective for electrochemical oxidation-induced Friedel–Crafts-type amidomethylation (path d in Scheme 1) [22–23]. We also succeeded in generating *N*-acyliminium ions from *N,N*-dimethylformamide (DMF) used as a solvent in the electrochemical carboxylation of benzyl bromides. Electrolysis of benzyl bromides in DMF containing 0.1 M Bu_4_NBF_4_ and iPr_2_NEt (1 equiv) using an undivided cell equipped with a Pt plate cathode and a Pt wire anode (a quasi-divided cell) [24–28] in the presence of carbon dioxide resulted in reductive carboxylation at the cathode and selective formation of *N*-acyliminium ions of DMF at the anode to produce coupling products, *N*-phenylacetoxymethyl-*N*-methylformamides, in good yields [29]. In this reaction system, the use of a quasi-divided cell enabled DMF to be oxidized with high selectivity at the anode even in the presence of carboxylate and bromide ions, which would generally be oxidized more easily than DMF. Accordingly, we tried this electrolysis system using a quasi-divided cell to apply electrochemical Friedel–Crafts-type amidomethylation of arenes, and we found that the use of iPr_2_NHEtBF_4_ in electrolysis using a quasi-divided cell was highly effective for electrochemical Friedel–Crafts-type amidomethylation of electron-rich arenes, such as 1,3,5-trimethoxybenzene and indoles (this work in Scheme 1). To the best of our knowledge, this is the first example of the use of trialkylammonium salts, such as iPr_2_NHEtBF_4_, in electroorganic synthesis, especially with the electrochemical oxidation system as a supporting electrolyte as well as a proton source for the cathodic reduction producing hydrogen gas. We report electrochemical Friedel–Crafts-type amidomethylation of electron-rich arenes by a novel electrochemical oxidation system using a quasi-divided cell and iPr_2_NHEtBF_4_.

## Results and Discussion

We chose 1,3,5-trimethoxybenzene (**1**) as a model substrate for electrochemical Friedel–Crafts-type amidomethylation. For electrolysis, a test tube-like undivided cell equipped with a Pt plate cathode (2 × 2 cm^2^) and a Pt wire anode (2 cm × 1 mm Ø) was used. Electrolysis of an *N,N*-dimethylacetamide (DMA) solution of **1** containing 0.1 M Bu_4_NBF_4_ as a supporting electrolyte, trifluoroacetic acid (TFA, 1 equiv) as a proton source for the cathodic reduction, and iPr_2_NEt (1 equiv) as a base for the formation of *N*-acyliminium ions of DMA at the anode was carried out under constant current conditions (20 mA/cm^2^) with 3 F/mol of electricity at 0 °C. It was found that 66% of **1** remained unchanged and mono-amidomethylation product **2** was formed in 16% yield along with 6% of di-substituted product **3** by analysis of the ^1^H NMR spectrum of the crude product mixture using 1,4-dinitrobenzene as an internal standard (Table 1, entry 1). These results indicate that anodic oxidation of not the substrate **1** but DMA successfully proceeded at the anode. In other words, *N*-acyliminium ions of DMA would expectedly be formed. We speculated that the reason for the lower product yields of **2** and **3** was a side reaction of the produced *N*-acyliminium ions with other nucleophiles in the reaction medium. The most likely nucleophile in this reaction medium was the trifluoroacetate ion, which was produced by electrochemical reduction of TFA at the cathode, although we could not detect the coupling product of trifluoroacetate and the corresponding *N*-acyliminium ion due to the high solubility in water. In addition to TFA, iPr_2_NEt was also included in this reaction medium, and they probably formed the corresponding ammonium trifluoroacetate in the reaction medium. The thus-generated trifluoroacetate ion could also react with *N*-acyliminium ions of DMA. Therefore, to avoid the reaction of the cathodic product with *N*-acyliminium ions, a proton source for which the conjugate base has no nucleophilicity would be necessary in the cathodic reduction. After several attempts, we finally reached HBF_4_·OEt_2_ as a proton source for cathodic reduction and the result is shown in entry 2 of Table 1. Strong increases of conversion of **1** and yield of **2** were observed. These results indicate that DMA was selectively oxidized at the anode to generate the corresponding *N*-acyliminium ions, which were trapped by trifluoroacetate ions preventing the desired amidomethylation in the presence of TFA. The exchange of the proton source from TFA to HBF_4_·OEt, for which the conjugate base has no nucleophilicity, improved the yield of **2** and the conversion of **1**.

**Table 1:** Effect of the proton source in electrochemical amidomethylation.

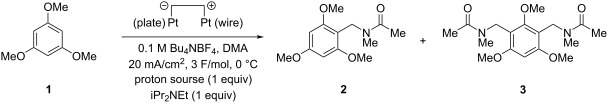				

Entry	Proton source	Conversion of **1** [%]^a^	Yield of **2** [%]^a^	Yield of **3** [%]^a^

1	CF_3_CO_2_H	34	16	6
2	HBF_4_·OEt_2_	80	63	12

^a^Determined by ^1^H NMR using 1,4-dinitrobenzene as an internal standard.

Incidentally, this reaction medium includes a base, iPr_2_NEt, that would accelerate the deprotonation step in the formation of *N*-acyliminium ions from DMA at the anode. TFA and HBF_4_·OEt_2_ will react with iPr_2_NEt in the reaction medium to form the corresponding ammonium salt. We thought that if trialkylammonium tetrafluoroborate, R_3_NHBF_4_, would be usable not only as a proton source for cathodic reduction but also as a supporting electrolyte, a novel and innovative electrochemical oxidation system could be developed. The use of R_3_NHBF_4_ in the electrochemical reaction as a supporting electrolyte and a proton source was investigated and the results are summarized in Table 2.

**Table 2:** Effect of trialkylammonium salt in electrochemical amidomethylation.

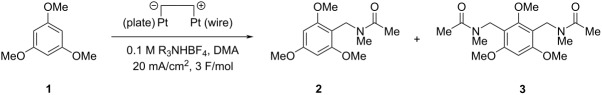					

Entry	R_3_NHBF_4_	Temperature [°C]	Conversion of **1** [%]^a^	Yield of **2** [%]^a^	Yield of **3** [%]^a^

1	Bu_3_NHBF_4_	0	83	62	12
2	Et_3_NHBF_4_	0	83	61	17
3	Et_3_NHBF_4_	−10	79	65	14
4	iPr_2_NHEtBF_4_	−10	82	72	6

^a^Determined by ^1^H NMR using 1,4-dinitrobenzene as an internal standard.

When electrolysis of **1** in DMA was carried out in the presence of 0.1 M Bu_3_NHBF_4_ without any other supporting electrolyte using a quasi-divided cell, the desired amidomethylation took place efficiently to give **2** in 62% ^1^H NMR yield along with **3** (Table 2, entry 1). The use of Et_3_NHBF_4_ instead of Bu_3_NHBF_4_ gave a similar result (Table 2, entry 2). A slight increase in the yield of **2** was observed when electrolysis of **1** was carried out using Et_3_NHBF_4_ at −10 °C (Table 2, entry 3). Instead of Et_3_NHBF_4_, sterically more hindered iPr_2_NHEtBF_4_ was effective for the electrochemical synthesis to give the desired compound **2** in the highest yield, 72% by ^1^H NMR (Table 2, entry 4). These results strongly indicate that trialkylammonium salts, R_3_NHBF_4_, can play roles not only as a proton source but also as supporting electrolyte in the electrochemical oxidation system using a quasi-divided cell. With these results in hand, we moved to screening of electrolysis conditions and the results are summarized in Table 3.

**Table 3:** Screening of reaction conditions in electrochemical amidomethylation.

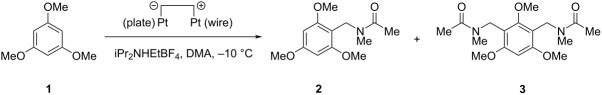						

Entry	Concentration of iPr_2_NHEtBF_4_ [M]	Current density [mA/cm^2^]	Electricity [F/mol]	Conversion of **1** [%]^a^	Yield of **2** [%]^a^	Yield of **3** [%]^a^

1	0.05	10	3	77	70	6
2	0.05	20	3	81	73	8
3	0.05	30	3	77	68	9
4	0.05	20	4	89	79	10
5	0.05	20	2	58	53	4
6	0.05	20	5	93	74	15
7	0.10	20	3	82	72	6
8	0.10	20	4	95	79 (71)^b^	15
9^c^	0.10	20	4	76	38	trace

^a^Determined by ^1^H NMR using 1,4-dinitrobenzene as an internal standard; ^b^Isolated yield; ^c^A Pt plate (2 × 2 cm^2^) was used as an anode.

Electrochemical Friedel–Crafts-type amidoalkylation also took place efficiently with a lower concentration (0.05 M) of the supporting electrolyte iPr_2_NHEtBF_4_, to give **2** in good yields under various electrolysis conditions (Table 3, entries 1–6). After several attempts in screening of current density (Table 3, entries 1–3), electricity (Table 3, entries 2 and 4–6), and the effect of concentration of the supporting electrolyte, the best result was obtained by a constant current electrolysis of **1** in DMA containing 0.05 or 0.1 M iPr_2_NHEtBF_4_ with 4 F/mol of electricity at −10 °C to yield amidomethylation product **2** in 79% ^1^H NMR yield (Table 3, entries 4 and 8) and 71% isolated yield (Table 3, entry 8). The use of a Pt plate (2 × 2 cm^2^) instead of a Pt wire as the anode resulted in a drastic decrease in the yield of **2** (Table 3, entry 9). These results indicate that a Pt wire anode plays an important role and that the use of a Pt wire as an anode is critical and essential in the present electrochemical amidomethylation.

**Scheme 2:**
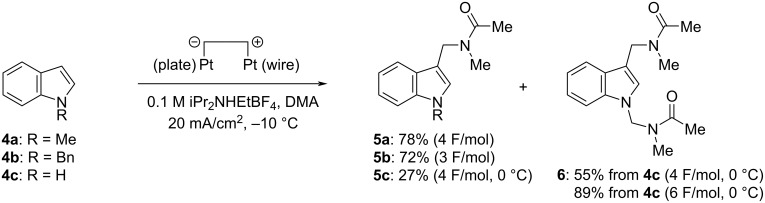
Electrochemical amidomethylation of indoles **4** in DMA.

**Scheme 3:**
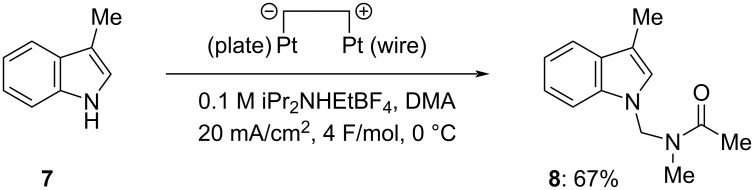
Electrochemical amidomethylation of 3-methyl-1*H*-indole (**7**) in DMA.

When other substituted benzenes such as anisole, 1,2- and 1,4-dimethoxybenzenes, 1,2,3-trimethoxybenzene, and 1,3,5-trimethylbenzene were used as substrates in the electrochemical Friedel–Crafts-type amidoalkylation, the desired products were not obtained/detected by ^1^H NMR. In contrast, it was reported that anisole [9,11,23] and 1,3,5-trimethylbenzene [23] could react with acyliminium ions generated by the chemical [9,11] or cation pool method [23] to produce amidomethylated products. These results indicate that the present electrochemical amidomethylation seems to be relatively less reactive than other chemical methods and the cation pool method. On the other hand, similar electrolysis of 1,3-dimethoxybenzene gave a mixture of products including regioisomeric mono-amidomethylation products together with diamidomethylation products, and it was difficult to analyze them exactly. Although *N*-acetylindole was also ineffective, several indoles were found to be applicable to the present amidomethylation reaction and the results are summarized in Scheme 2. When *N*-methylindole (**4a**) was electrolyzed using a quasi-divided cell under the conditions shown in Scheme 1, electrochemical amidomethylation took place efficiently at the C3 position of **4a** to yield **5a** in 78% isolated yield. Similar electrolysis of *N*-benzylindole (**4b**) also induced amidomethylation at its C3 position to give **5b** in 72% isolated yield. To our surprise, we found that electrolysis of *N*-benzylindole (**4b**) at −10 °C under the conditions of 20 mA/cm^2^ of current density and a lower concentration (0.05 M) of iPr_2_NHEtBF_4_ in DMA with 3-6 F/mol of electricity resulted in removal of the benzyl group followed by amidomethylation at the nitrogen atom of **4b** to yield *N*,3-diamidomethylated indole **6** in 4–34% (^1^H NMR yield), although similar electrolysis with 2 F/mol of electricity gave only **5b** in 66% yield with 68% conversion. It is thought that supplying an excess amount of electricity under the conditions of a lower concentration of the proton source (supporting electrolyte), iPr_2_NHEtBF_4_, caused competitive electrochemical reduction of a proton and the *N*-benzyl group of **5b** at the cathode. We also carried out electrochemical amidomethylation of indole (**4c**) and found that a mixture of 3-amidomethylated indole **5c** and *N*,3-diamidomethylated indole **6** was produced. However, *N*-amidomethylated indole was not observed in the ^1^H NMR spectra of the crude products. These results indicate that amidomethylation firstly occurs at the C3 position of **4c** and then the second amidomethylation takes place on the indole nitrogen atom of **5c**. Despite our efforts, selective formation of **5c** could not be achieved under various electrolysis conditions. Electrolysis of **4c** supplying 4 F/mol of electricity at 0 °C afforded 3-amidomethylated **5c** and *N*,3-diamidomethylated **6** in 27% and 55% isolated yields, respectively. Fortunately, electrolysis with 6 F/mol of electricity could predominantly produce diamidomethylated **6** in 89% isolated yield. Similar amidomethylation of indole (**4c**) using chemical methods has already been reported by Shirakawa [12] and Doan [11]. However, mono-amidomethylation at the C3 position of indole **4c** only took place to yield **5c** predominantly and no *N*-amidomethylated product was obtained. We investigated electrochemical amidomethylation of 3-methylindole (**7**) and found that amidomethylation similarly proceeded at the nitrogen atom of **7** to yield *N*-amidomethylated **8** in 67% isolated yield (Scheme 3). These results indicate that the present electrochemical amidomethylation has quite different reactivity from that of the reported chemical ones, although the exact reason is not clear at the present.

**Scheme 4:**
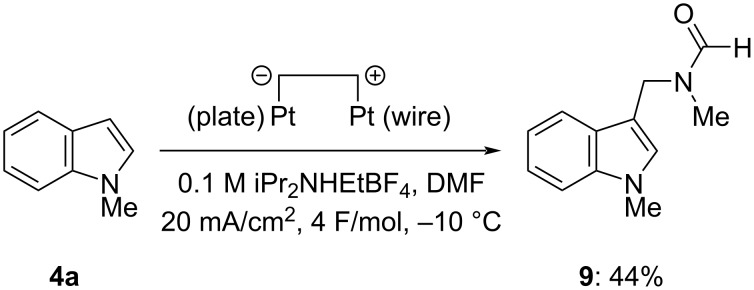
Electrochemical amidomethylation of *N*-methyl-1*H*-indole (**4a**) in DMF.

It was found that DMF instead of DMA was also applicable to the present electrochemical amidomethylation. Similar electrolysis of **4a** in DMF containing 0.1 M iPr_2_NHEtBF_4_ using a quasi-divided cell gave 3-amidomethylated *N*-methylindole **9** in 47% ^1^H NMR yield and 44% isolated yield at full conversion (Scheme 4). The moderate yield of **9** is thought to be due to its high solubility in water.

**Scheme 5:**
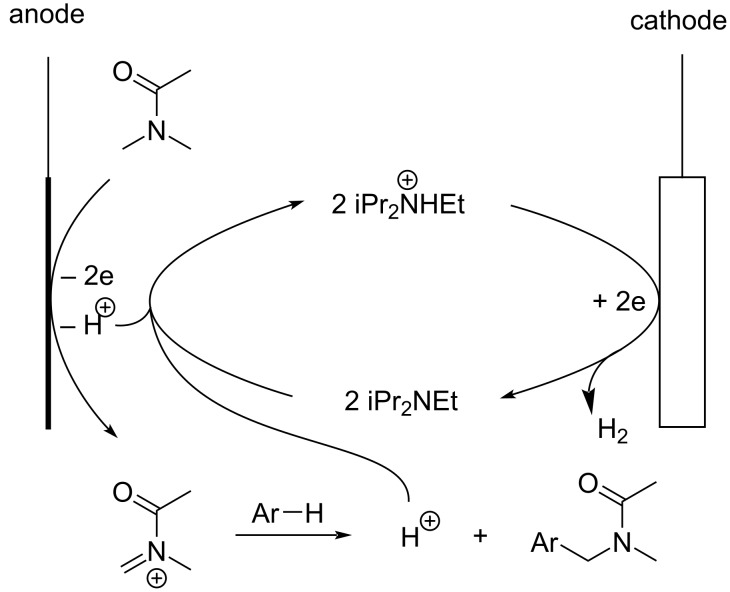
Probable reaction pathway of the electrochemical amidomethylation.

A probable reaction pathway is shown in Scheme 5. The present amidomethylation is induced by electrolysis using a quasi-divided cell equipped with a Pt plate cathode (2 × 2 cm^2^) and a Pt wire anode (2 cm × 1 mm Ø) in DMA containing 0.1 M iPr_2_NHEtBF_4_. At the cathode, electrochemical reduction of a proton in iPr_2_NHEt^+^ takes place to generate hydrogen gas and iPr_2_NEt. Evolution of a gas at the cathode can be observed visually. It is well known that electrochemical reduction can generate the intermediates/products which play as bases. Thus-generated bases are called electrogenerated bases (EGBs) and have widely been used in electroorganic synthesis [30–35]. At the anode, electrochemical one-electron oxidation of the solvent, DMA, takes place selectively. Deprotonation, probably supported by iPr_2_NEt generated at the cathode, followed by further one-electron oxidation generates the corresponding *N*-acyliminium ion of DMA. Deprotonation supported by iPr_2_NEt produces iPr_2_NHEt^+^, which is used again as a proton source and a supporting electrolyte. In a quasi-divided cell, two electrodes have largely different surface areas. Current density, 20 mA/cm^2^, is realized at a Pt plate cathode. On the other hand, the anode is a Pt wire, which has a significantly smaller surface area, and a much higher current density is realized. At the anode with a much higher current density, the concentrations of the starting material, iPr_2_NEt, and the products, which seem to be more easily oxidized, are relatively low and there is insufficient mass transfer at the anode that results in selective oxidation of the solvent, DMA, which is the substance with the largest amount at the anode [28–29].

## Conclusion

We have developed a novel electrochemical oxidation system using a quasi-divided cell and trialkylammonium tetrafluoroborates, especially iPr_2_NHEtBF_4_, both as a supporting electrolyte and as a proton source for the cathodic reduction. The system was successfully applied to Friedel–Crafts-type electrochemical amidoalkylation of arenes, such as 1,3,5-trimethoxybenzene and indoles, to yield the corresponding amidomethylated products in good to high yields. The novel electrochemical oxidation system will be promising as a powerful tool for electroorganic synthesis using anodic oxidation. In addition, trialkylammonium salts have high potential both as novel supporting electrolytes and proton sources for cathodic reduction in the anodic oxidation process.

## Supporting Information

File 1 General experimental information, preparation of trialkylammonium salts, general procedure for electrolysis, spectral data information including ^1^H and ^13^C NMR spectra of new compounds.
