# Supplementary material for: Electrochemical Friedel–Crafts-type amidomethylation of arenes by a novel electrochemical oxidation system using a quasi-divided cell and trialkylammonium tetrafluoroborate
Source: Beilstein J Org Chem. 2022 Aug 18;18:1040–6. doi: 10.3762/bjoc.18.105 (PMC9443422; doi:10.3762/bjoc.18.105)
Supplement: File 1 — General experimental information, preparation of trialkylammonium salts, general procedure for electrolysis, spectral data information including 1H and 13C NMR spectra of new compounds. [file Beilstein_J_Org_Chem-18-1040-s001.pdf]

BEILSTEIN JOURNAL OF ORGANIC CHEMISTRY

Supporting Information

for

Electrochemical Friedel–Crafts-type amidomethylation of arenes by a novel electrochemical oxidation system using a quasi-divided cell and trialkylammonium tetrafluoroborate

Hisanori Senboku, Mizuki Hayama and Hidetoshi Matsuno

Beilstein J. Org. Chem. **2022,** *18,* 1040–1046. doi:10.3762/bjoc.18.105

General experimental information, preparation of trialkylammonium salts, general procedure for electrolysis, spectral data information including ^{1}H and ^{13}C NMR spectra of new compounds

License and Terms: This is a supporting information file under the terms of the Creative Commons Attribution License (https://creativecommons.org/licenses/by/4.0). Please note that the reuse, redistribution and reproduction in particular requires that the author(s) and source are credited and that individual graphics may be subject to special legal provisions.
The license is subject to the *Beilstein Journal of Organic Chemistry* terms and conditions: (https://www.beilstein-journals.org/bjoc/terms)

Table of Contents:

1. General information………S2
2. Preparation of trialkylammonium tetrafluoroborates……………………………………………………………………..S2
3. General procedure for electrochemical amidomethylaion of **1**, **4**, or **7**……………………………………………………..S2
4. Spectral data of the products, **2**, **5**, **6**, **8**, and **9**………………………………………………………………………………..S3
5. References……..S5
6. ^{1}H and ^{13}C Spectra of new compounds, **5b**, **6**, and **8**. ……………………………………………………………………..S6

1. General information:

^1H (400 MHz) and ^{13}C (100 MHz) NMR spectra were recorded in $CDCl_3$ or DMSO-d_6 with a JEOL JNM-ECS400 FT NMR spectrometer. The chemical shifts, δ, are given in ppm with tetramethylsilane (δ 0 ppm) or DMSO (δ 2.50 ppm) for ^1H and $CDCl_3$ (δ 77.0 ppm) or DMSO-d_6 (δ 39.5 ppm) for ^{13}C as internal references. *J* values are in Hz. Peak multiplicities were given as follows: s, singlet; d, doublet; t, triplet; q, quartet; sext, sextet; m, multiplet; br, broad. In ^1H and ^{13}C NMR, signals of a minor rotamer are given in parentheses. HRMS (ESI) spectra were measured with a Thermo Scientific Exactive at the Instrumental Analysis Division, Global Facility Center, Creative Research Institution, Hokkaido University. Melting points were measured on a Yanagimoto micro melting point apparatus and are uncorrected. Reagents and solvents including anhydrous DMA and anhydrous DMF were commercially available and were used as received without further purification. Electrochemical reactions were carried out using a Constant Current Power Supply (model 5944), Metronix Corp., Tokyo.

Arenes as substrates, 1,3,5-trimethoxybenzene (**1**) and indoles **4** and **7** except **4b** are commercially available as well as anisole, 1,2- and 1,4-dimethoxybenzenes, 1,2,3-trimethoxybenzene, and 1,3,5-trimethylbenzene. *N*-benzyl-1*H*-indole (**4b**)[1)] and *N*-acetyl-1*H*-indole[2)] were prepared from 1*H*-indole (**4c**) according to the reported procedures.[1,2)]

2. Preparation of trialkylammonium tetrafluoroborates (R_3NHBF_4):

To a solution of an appropriate amine (40 mmol) in ether (60 mL) was dropwise added $HBF_4 \cdot OEt_2$ (40 mmol, 5.5 mL) for 5 min. After the addition, the reaction mixture was stirred for 1 h at 0 °C, and then the mixture was stirred at room temperature for further 30 min. The solid was collected by filtration with suction and was recrystallized from ethyl acetate (Et_3NHBF_4 and Bu_3NHBF_4) or methanol ($iPr_2NHEtBF_4$) to give trialkylammonium salt, which was used after dryness under reduced pressure.

All trialkylammonium salts, Et_3NHBF_4,[3)] Bu_3NHBF_4,[4)] and $iPr_2NHEtBF_4$[5)] are known compounds, although ^1H and ^{13}C spectra of Bu_3NHBF_4 have not been reported.

Tributylammonium tetrafluoroborates (Bu_3NHBF_4): ^1H NMR (400 MHz, $CDCl_3$): δ 0.98 (9H, t, *J* = 7.8 Hz), 1.41 (6H, sext, *J* = 7.8 Hz), 1.67-1.74 (6H, m), 3.10-3.16 (6H, m), 7.08 (1H, br. s). ^{13}C NMR (100 MHz, $CDCl_3$): δ 13.4, 19.6, 25.5, 53.5.

3. General procedure for electrochemical amidomethylaion of 1, 4, or 7.

A solution of arene **1**, **4** or **7** (1 mmol) in anhydrous DMA (10 mL) containing 0.1 M $iPr_2NHEtBF_4$ (217 mg, 1 mmol) was electrolyzed under a nitrogen atmosphere at −10 °C with a constant current (20 mA cm^{-2}). A test tube-like (ca. 25 mm Ø) undivided cell equipped with a Pt plate cathode (2 × 2 cm^2) and a Pt wire anode (2 cm, 1 mm Ø) was used for the electrolysis. After an appropriate electricity was supplied, the solvent was evaporated. To the residue was added saturated aqueous NH_4Cl (20 mL), and then the mixture was extracted with ethyl acetate (20 mL × 5). The combined organic layer was washed with brine (100 mL) and was dried over anhydrous $MgSO_4$. Evaporation of the solvent gave a crude product, which was analyzed by ^1H NMR in $CDCl_3$ with the addition of an appropriate amount of 1,4-dinitrobenzene as an internal standard. Isolation/purification of the product was carried out by column chromatography on silica gel.

The products **2**,[6-9)] **3**,[6,7,9)] **5a**,[6,8,10,11)] **5c**,[6,8,10,12)] and **9**[7)] are known compounds (**3** was not isolated in this paper) and their spectral data were good agreement with the reported values in every respect.

4. Spectral data of the products, 2, 5, 6, 8, and 9.

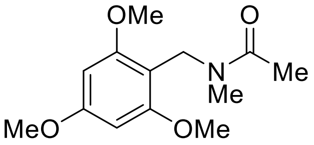

N-Methyl-*N*-[(2,4,6-trimethoxyphenyl)methyl]acetamide (**2**): ^{1}H NMR (400 MHz, $CDCl_3$): δ 2.28 (2.06) (3H, s), 2.70 (3H, s), 3.78, 3.79, 3.81 (9H, s), 4.44 (4.62) (2H, s), 6.11 (2H, s). ^{13}C NMR (100 MHz, $CDCl_3$): δ 21.3 (22.1), 31.1 (33.7), 42.1 (38.0), 55.2, (55.3), (55.4), 55.6, 90.1, 104.7 (105.1), 159.6 (160.0), 161.1 (160.8), 170.9 (170.8).

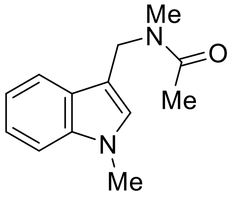

N-Methyl-*N*-[(1-methyl-1*H*-indol-3-yl)methyl]acetamide (**5a**): ^{1}H NMR (400 MHz, $CDCl_3$): δ 2.10 (2.26) (3H, s), 2.91 (2.97) (3H, s), 3.76 (3.77) (3H, s), 4.72 (4.67) (2H, s), 7.04 (6.91) (1H, s), 7.10-7.16 (1H, m), 7.22-7.34 (m, 2H), 7.69 (7.51) (1H, br d, *J* = 8.2 Hz). ^{13}C NMR (100 MHz, $CDCl_3$): δ 21.9 (21.4), 32.5 (33.1), 34.9 (32.7), 41.3 (46.5), 109.0 (109.4), 110.4 (109.8), 119.2 (118.4), 119.4 (119.3), 121.7 (121.9), 126.6 (126.4), 128.5 (127.2), 136.8 (137.1), 170.1 (170.6).

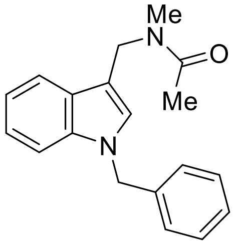

N-Methyl-*N*-[(1-phenylmethyl-1*H*-indol-3-yl)methyl]acetamide (**5b**): Brown solid, mp 92.5-96.5 °C. ^{1}H NMR (400 MHz, $CDCl_3$): δ 2.11 (2.26) (3H, s), 2.90 (2.97) (3H, s), 4.75 (4.69) (2H, s), 5.29 (5.30) (2H, s), 6.99-7.34 (9H, m), 7.72 (7.54) (1H, d, *J* = 8.2 Hz). ^{13}C NMR (100 MHz, $CDCl_3$): δ 22.0 (21.5), 34.8 (33.1), 41.3 (46.5), 49.8 (49.9), 109.6 (110.0), 111.2 (110.7), 119.5 (118.6), 119.7 (119.6), 122.0 (122.2), 126.56 (126.1), 126.63, 127.5 (127.6), 127.9, 128.6 (128.7), 136.6 (136.8), 137.2 (137.1), 170.1 (170.5). HRMS (ESI) calculated for $C_{19}H_{20}N_2NaO$ $[M + Na]^+$: 315.1468; found: 315.1468.

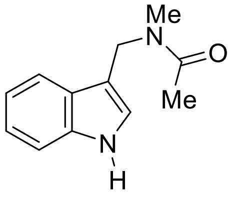

N-Methyl-*N*-[(1*H*-indol-3-yl)methyl]acetamide (**5c**): ^{1}H NMR (400 MHz, $CDCl_3$): δ 2.12 (2.27) (3H, s), 2.90 (2.98), (3H, s), 4.76 (4.69) (2H, s), 7.04-7.25 (3H, m), 7.37 (7.41) (1H, d, *J* = 8.2 Hz), 7.72 (7.53) (1H, d, *J* = 8.2 Hz), 8.48 (8.67) (1H, br s). ^{13}C NMR (100 MHz, $CDCl_3$): δ 22.0 (21.5), 34.9 (33.3), 41.6 (46.7), 111.2 (111.0), 111.5 (111.6), 119.2 (118.2), 119.5 (119.6), 122.0 (122.2), 124.1 (122.4), 126.7 (126.0), 136.4 (136.6), 170.5 (170.9).

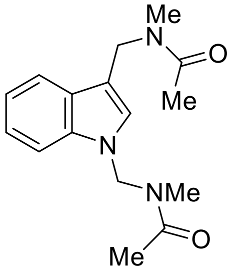

N,N'-(1*H*-Indole-1,3-diyl)bis(methylene)bis(*N*-methylacetamide) (**6**): Brown solid, mp 98.5-102.5 °C. ^{1}H NMR (400 MHz, $CDCl_3$): δ 2.08, 2.11 2.12, 2.26, 2.36 (6H, s), 2.90, 2.920, 2.928, 2.933, 2.94, 2.96, 3.01 (6H, s), 4.66, 4.72 (2H, s), 5.58, 5.60, 5.68, 5.69 (2H, s), 7.08-7.31 (3H, m), 7.51 (1H, br d, *J* = 8.2 Hz), 7.70 (7.57, 7.61) (1H, d, *J* = 8.2 Hz). ^{13}C NMR (100 MHz, $CDCl_3$): δ 21.4, 21.5, 21.6, 21.8, 21.9, 31.8, 33.0, 34.5, 34.8, 35.0, 37.9, 41.2, 46.3, 56.0, 56.2, 60.4, 60.5, 108.8, 109.8, 110.3, 111.3, 111.9, 113.1, 118.5, 119.6, 119.9, 120.1, 122.4, 122.7, 125.5, 126.3, 126.8, 127.5, 127.9, 136.2, 136.4, 169.8, 170.1, 170.5, 171.0. In ^{1}H and ^{13}C NMR spectra of **6** in $CDCl_3$ at rt, possible four rotamers were detected, and it is too difficult to analyze all of signals. Therefore, all of the detected signals are listed. We also tried ^{1}H NMR and ^{13}C NMR in DMSO-d_6 at 150 °C. ^{1}H NMR (400 MHz, DMSO-d_6, 150 °C): δ 2.08 (3H, s), 2.10 (3H, s), 2.886 (3H, s), 2.890 (3H, s), 4.66 (2H, s), 5.69 (2H, s), 7.07 (1H, br t, *J* = 7.8 Hz), 7.18 (1H, br t, *J* = 8.2 Hz), 7.34 (1H, s), 7.56 (1H, d, *J* = 8.2 Hz), 7.60 (1H, d, *J* = 7.8 Hz). ^{13}C NMR (100 MHz, DMSO-d_6, 150 °C): δ20.4 (overlapped), 32.9 (overlapped), 42.1, 56.5, 109.5, 111.2, 118.2, 118.8, 121.2, 126.83, 126.89, 135.9, 168.6, 169.6. In ^{13}C NMR in DMSO-d_6, signals thought to be derived from a major rotamer are only listed. HRMS (ESI) calculated for $C_{16}H_{21}N_3NaO_2$ $[M + Na]^+$: 310.1526; found: 310.1524.

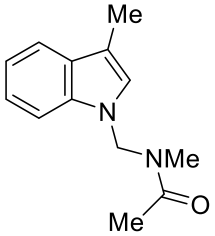

N-Methyl-*N*-[(3-methyl-1*H*-indol-1-yl)methyl]acetamide (**8**): Yellow oil. ^{1}H NMR (400 MHz, $CDCl_3$): δ 2.11 (2.35) (3H, s), 2.31 (d, *J* = 0.9 Hz) (2.32, br s) (3H), 2.90 (2.86) (3H, s), 5.66 (5.55) (2H, s), 7.00 (6.86), (1H, br s), 7.13 (7.16) (1H, br t, *J* = 7.3 Hz), 7.22 (7.25) (1H, br t, *J* = 7.3 Hz), 7.48 (7.30) (1H, d, *J* = 8.2 Hz), 7.56 (7.59) (1H, d, *J* = 8.2 Hz). ^{13}C NMR (100 MHz, $CDCl_3$): δ 9.5, 21.9 (21.6), 34.4 (31.8), 55.8 (60.2), 109.6 (108.6), 111.5 (112.7), 118.9 (119.3), 119.2 (119.5), 122.0 (122.3), 125.8 (123.4), 128.9 (129.1), 136.2, 171.0 (169.8). HRMS (ESI) calculated for $C_{13}H_{16}N_2NaO$ $[M + Na]^+$: 239.1155; found: 239.1152.

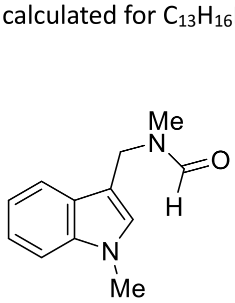

N-Methyl-*N*-[(1-methyl-1*H*-indol-3-yl)methyl]formamide (**9**): ^{1}H NMR (400 MHz, $CDCl_3$): δ 2.80 (2.82) (3H, s), 3.78 (3.77) (3H, s), 4.56 (4.67) (2H, s), 6.99 (7.05) (1H, s), 7.11-7.15 (1H, m), 7.22-7.34 (2H, m), 7.52 (7.68) (1H, d, *J* = 8.2 Hz), 8.40 (8.10) (1H, s). ^{13}C NMR (100 MHz, $CDCl_3$): δ 29.0, 32.65, 32.73, 33.8, 38.5, 45.1, 108.8, 109.2 (two signals overlapped), 109.5, 118.5, 119.29, 119.36, 119.5, 121.9, 122.1, 126.7, 127.1, 128.0, 128.7, 136.9, 137.2, 162.1, 162.4. In ^{13}CNMR data, all signals of a 53/47 mixture of two rotamers are listed without distinction due to its difficulty.

5. References:

1) Shi, Z.; Hammond, G. B.; Xu, B. *ACS Omega* **2018**, *3*, 6748–6756.

2) Benkovics, T.; Guzei, I. A.; Yoon, T. P. *Angew. Chem. Int. Ed*. **2010**, *49*, 9153–9157.

3) Guo, R.; Qi, X.; Xiang, H.; Geaneotes, P.; Wang, R.; Liu, P.; Wang, Y. -M. *Angew. Chem. Int. Ed.* **2020**, *59*, 16651–16660.

4) a) Zabinska, G.; Ferloni, P.; Sanesi, M. *Thermochim. Acta* **1988**, *137*, 39–49; b) Lennon, P.; Rosenblum, M. *J. Am. Chem. Soc.* **1983**, *105*, 1233–1241.

5) Andrews, R. S.; Becker, J. J.; Gagné, M. R. *Angew. Chem. Int. Ed.* **2010**, *49*, 7274–7276.

6) Shirakawa, E.; Uchiyama, N.; Hayashi, T. *J. Org. Chem.* **2011**, *76*, 25–34.

7) Dai, C.; Meschini, F.; Narayanam, J. M. R.; Stephenson, C. R. J. *J. Org. Chem.* **2012**, *77*, 4425−4431.

8) Nakamura, K.; Togo, H. *Eur. J. Org. Chem.* **2020**, 4713–4722.

9) Kaur, J.; Shahin, A.; Barham, J. P. *Org. Lett.* **2021**, *23*, 2002−2006.

10) Doan, S. H.; Nguyen, K. D.; Huynh, P. T.; Nguyen, T. T.; Phan, N. T. S. *J. Mol. Catal. A: Chemical* **2016**, *423*, 433–440.

11) Ikeda, K.; Morimoto, T.; Sekiya, M. *Chem. Pharm. Bull.* **1980**, *28*, 1178–1182.

12) Pedras, M. S. C.; Minic, Z.; Jha, M. *FEBS J.* **2008**, *275*, 3691–3705.

6. ^{1}H and ^{13}C spectra of new compounds 5b, 6, and 8.

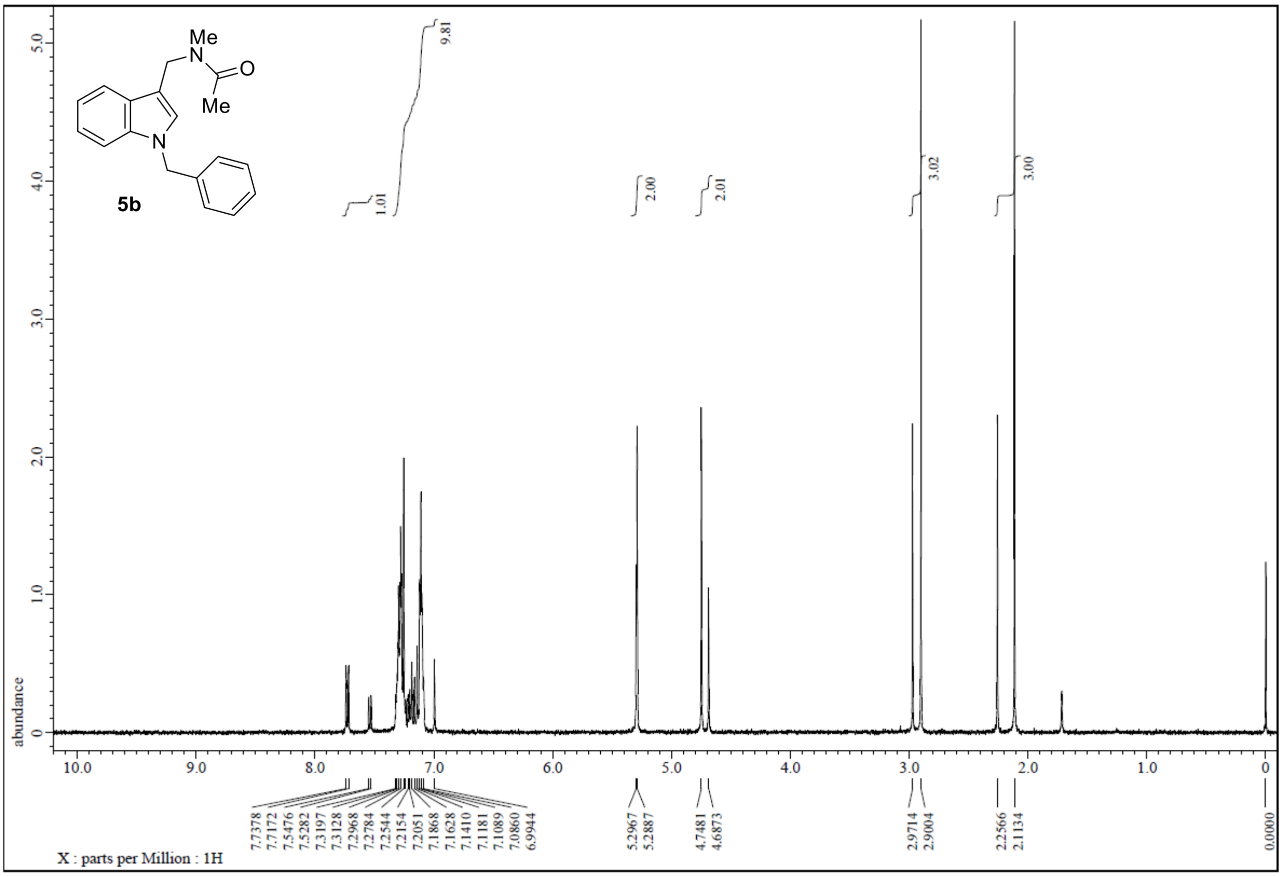

Fig. 1. ^{1}H NMR (400 MHz; $CDCl_3$) spectrum of *N*-methyl-*N*-[(1-phenylmethyl-1*H*-indol-3-yl)methyl]acetamide (**5b**).

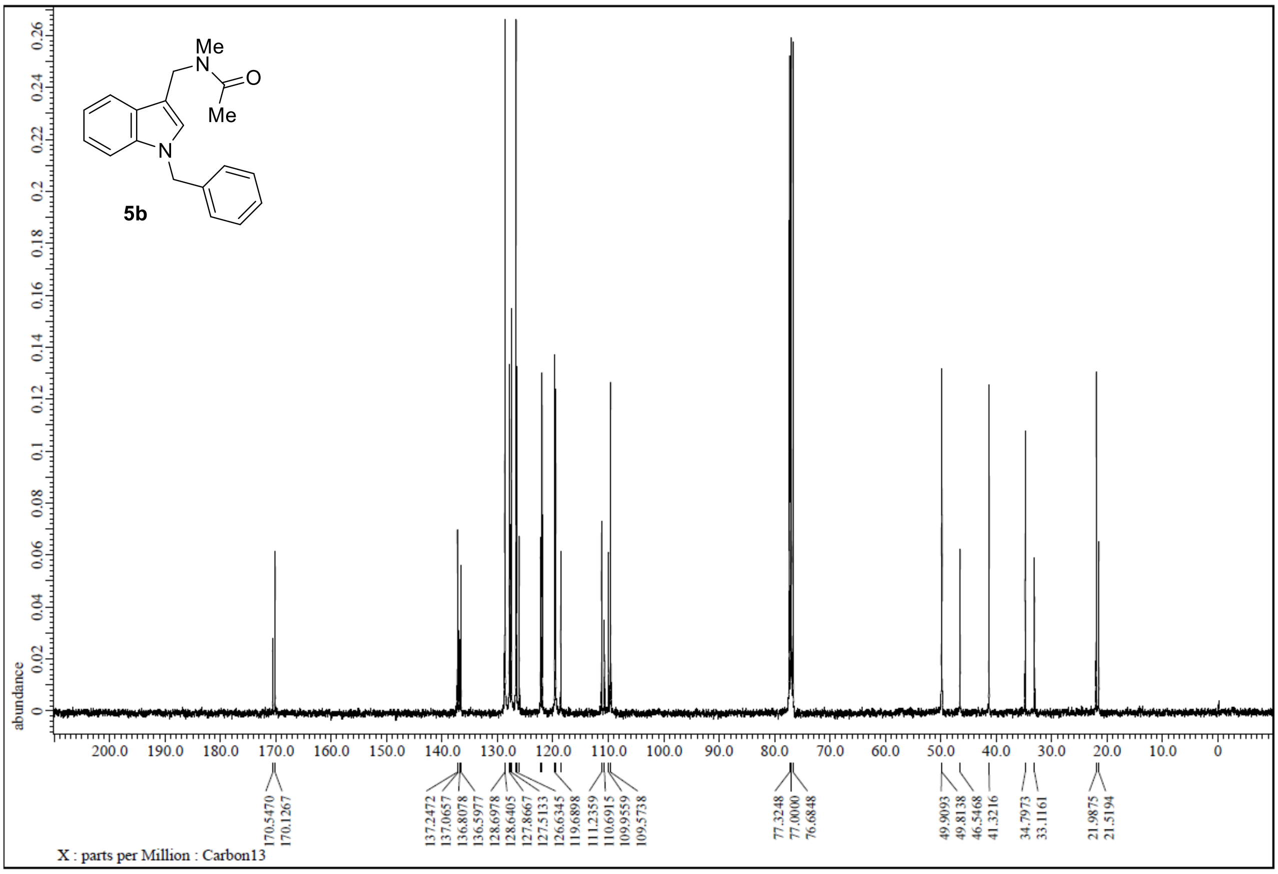

Fig. 2. ^{13}C NMR (100 MHz; $CDCl_3$) spectrum of *N*-methyl-*N*-[(1-phenylmethyl-1*H*-indol-3-yl)methyl]acetamide (**5b**).

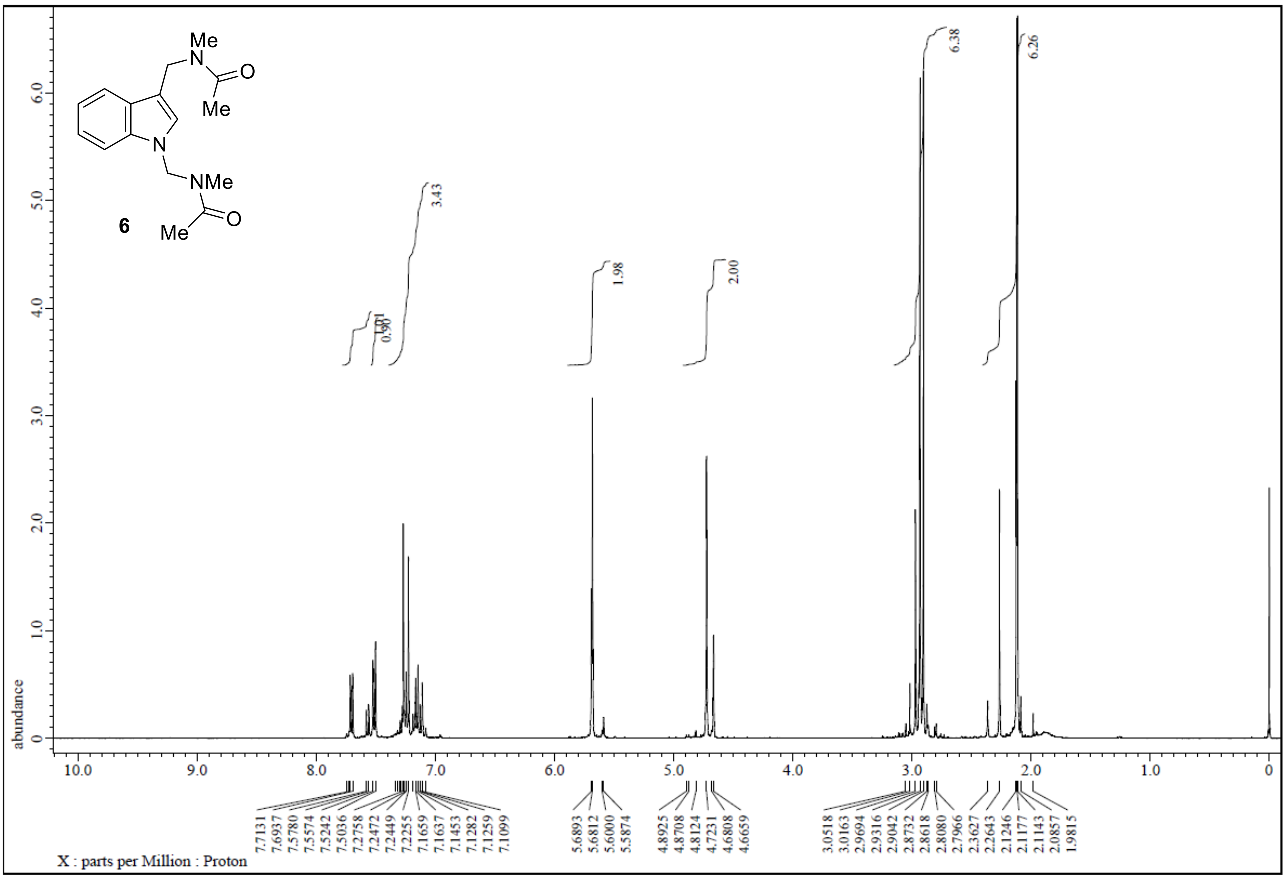

Fig. 3. ^{1}H NMR (400 MHz; $CDCl_3$) spectrum of *N,N'*-(1*H*-indole-1,3-diyl)bis(methylene)bis(*N*-methylacetamide) (**6**).

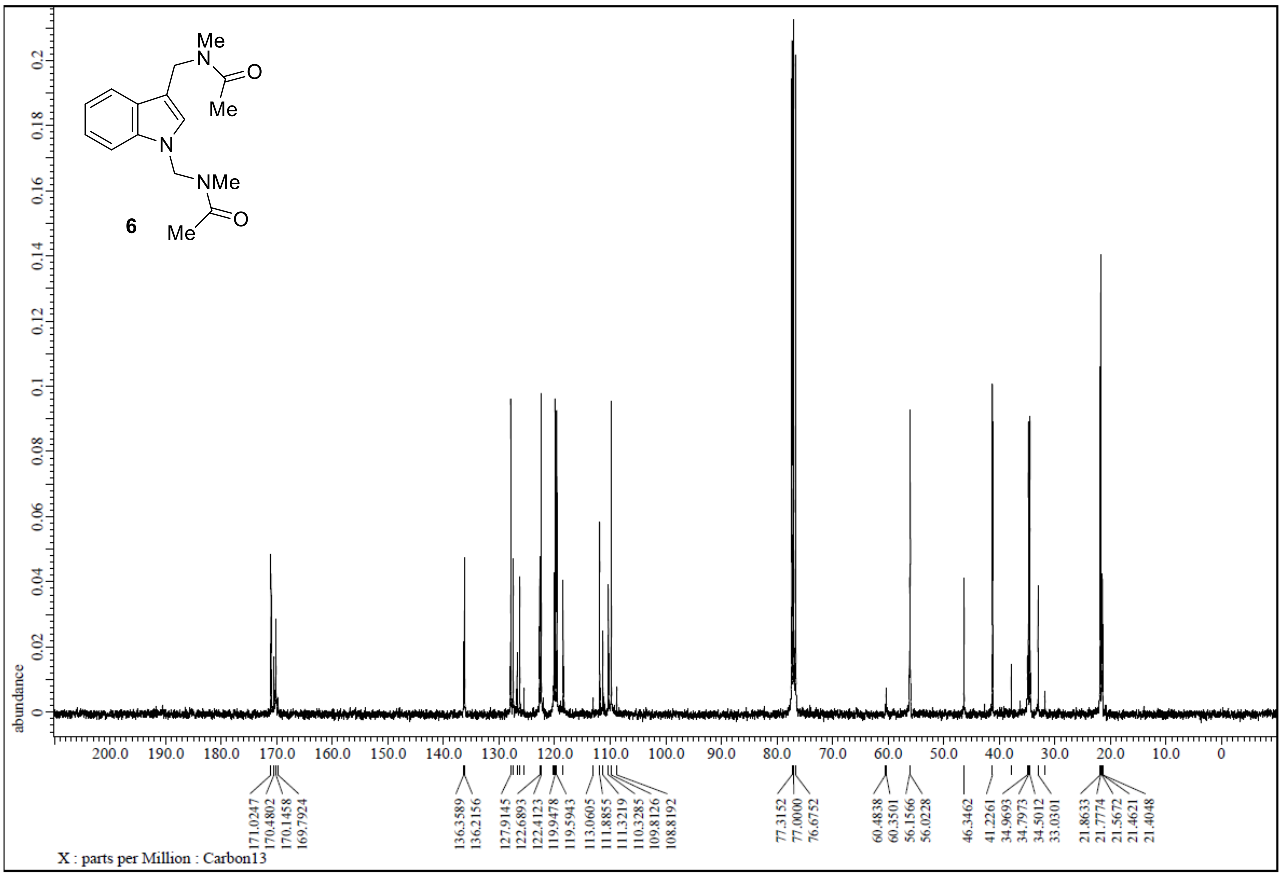

Fig. 4. ^{13}C NMR (100 MHz; $CDCl_3$) spectrum of *N,N'*-(1*H*-indole-1,3-diyl)bis(methylene)bis(*N*-methylacetamide) (**6**).

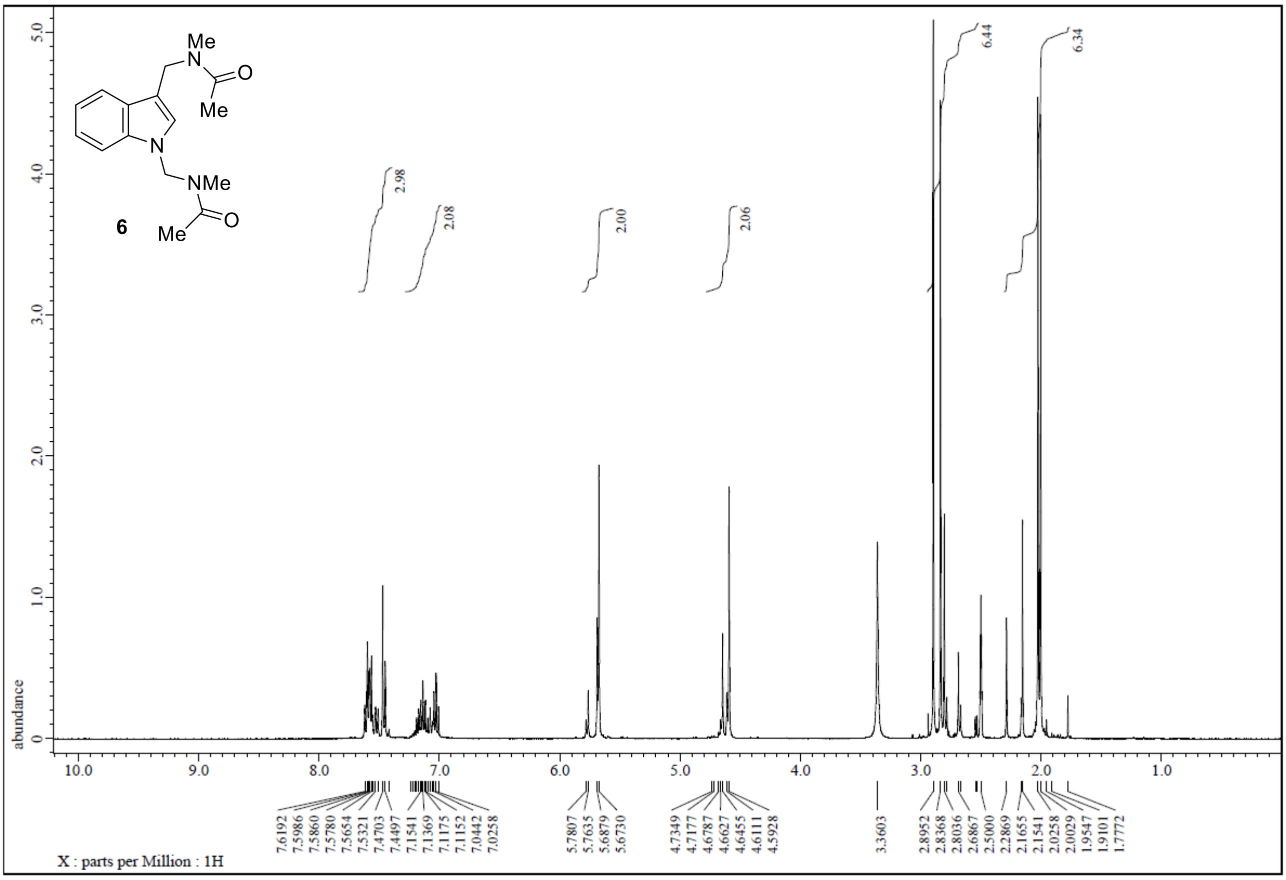

Fig. 5. ^{1}H NMR (400 MHz; DMSO-d_6, rt) spectrum of *N,N'*-(1*H*-indole-1,3-diyl)bis(methylene)bis(*N*-methylacetamide) (**6**).

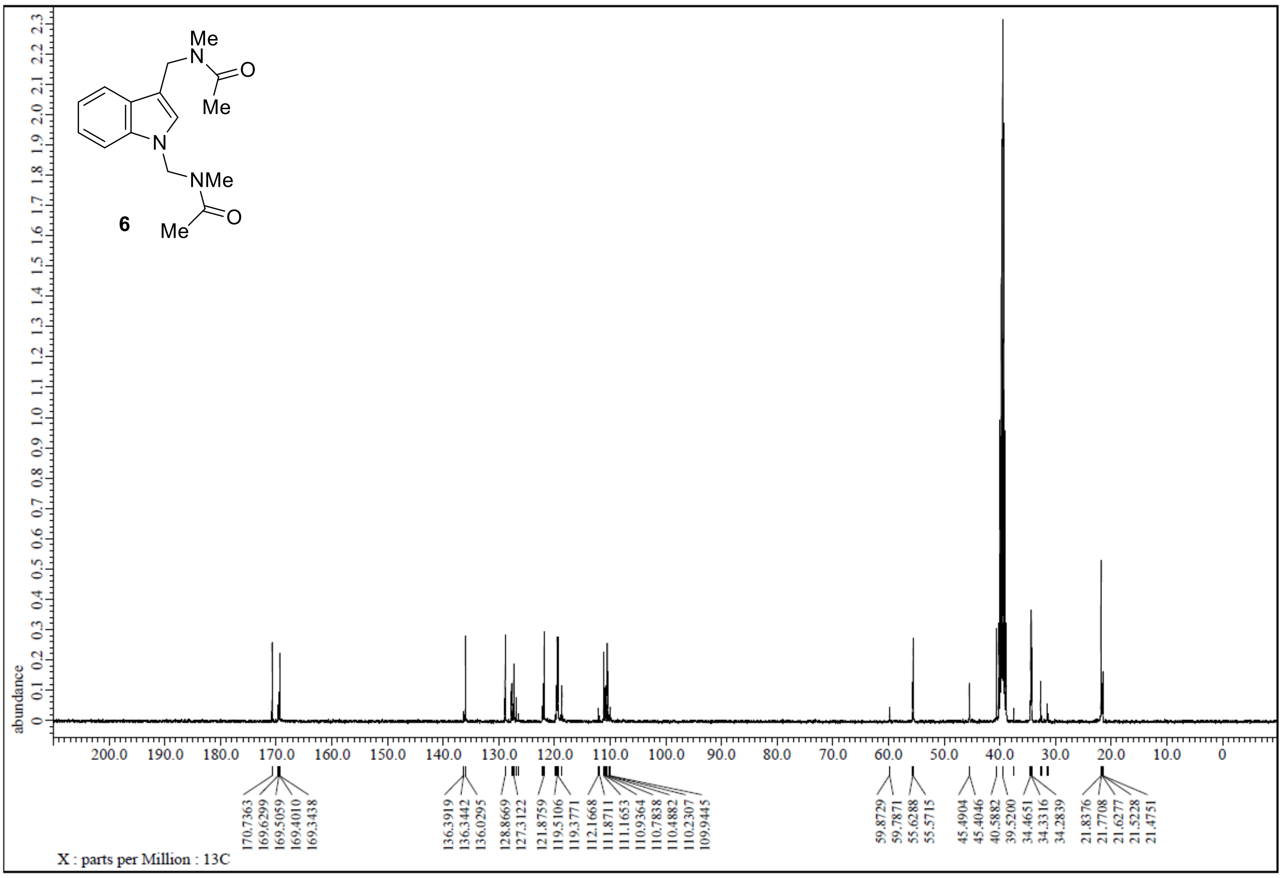

Fig. 6. ^{13}C NMR (100 MHz; DMSO-d_6, rt) spectrum of *N,N'*-(1*H*-indole-1,3-diyl)bis(methylene)bis(*N*-methylacetamide) (**6**).

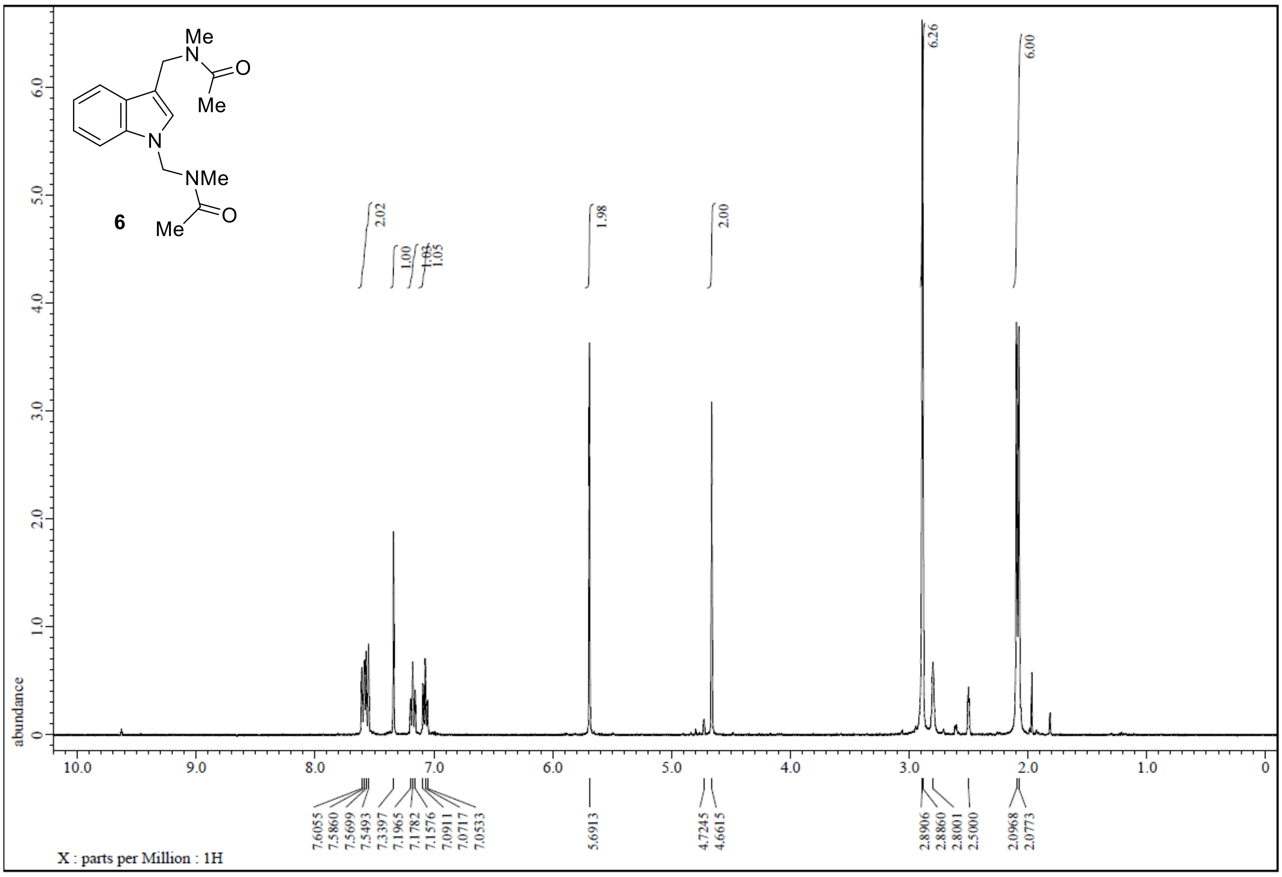

Fig. 7. ^{1}H NMR (400 MHz; DMSO-d_6, 150 °C) spectrum of *N,N'*-(1*H*-indole-1,3-diyl)bis(methylene)bis(*N*-methylacetamide) (**6**).

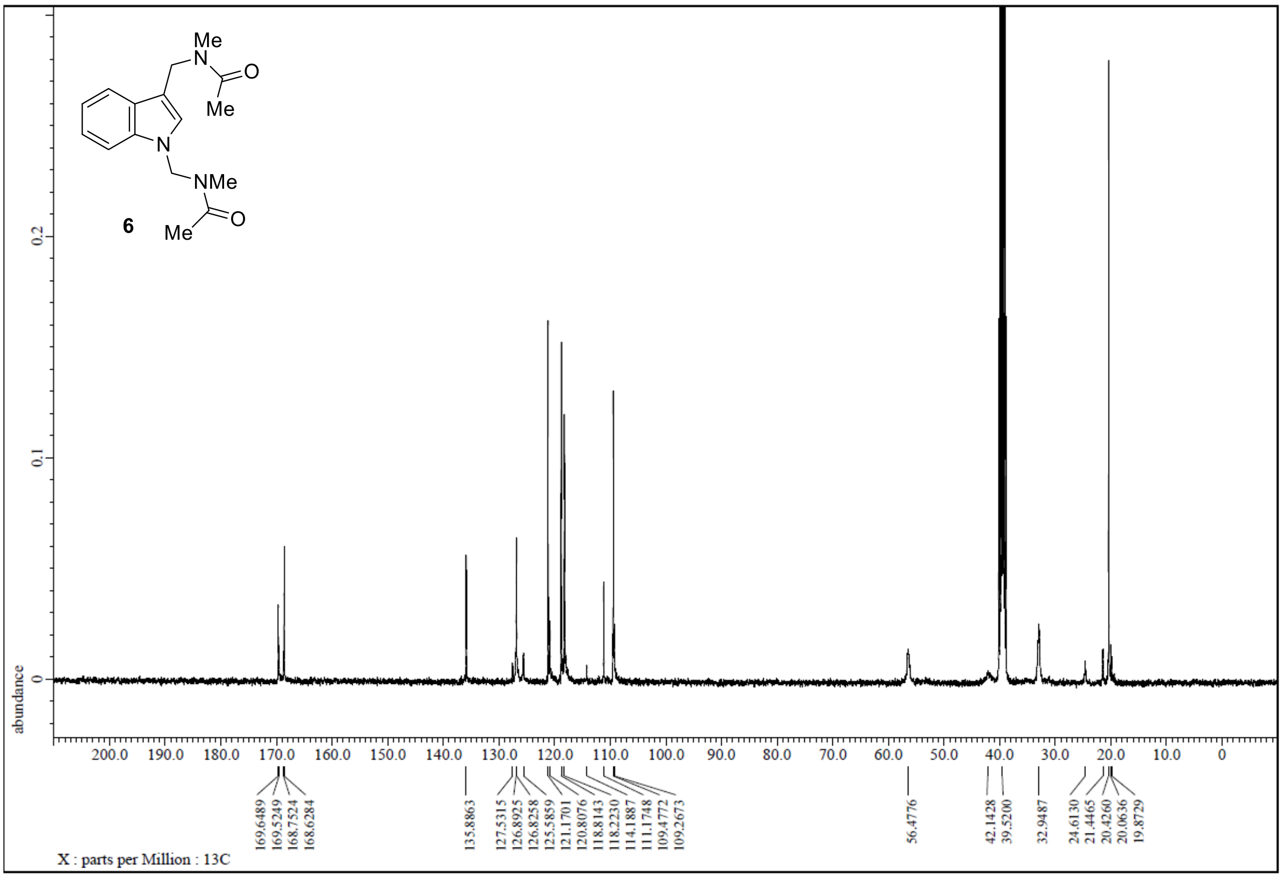

Fig. 8. ^{13}C NMR (100 MHz; DMSO-d_6, 150 °C) spectrum of *N,N'*-(1*H*-indole-1,3-diyl)bis(methylene)bis(*N*-methylacetamide) (**6**).

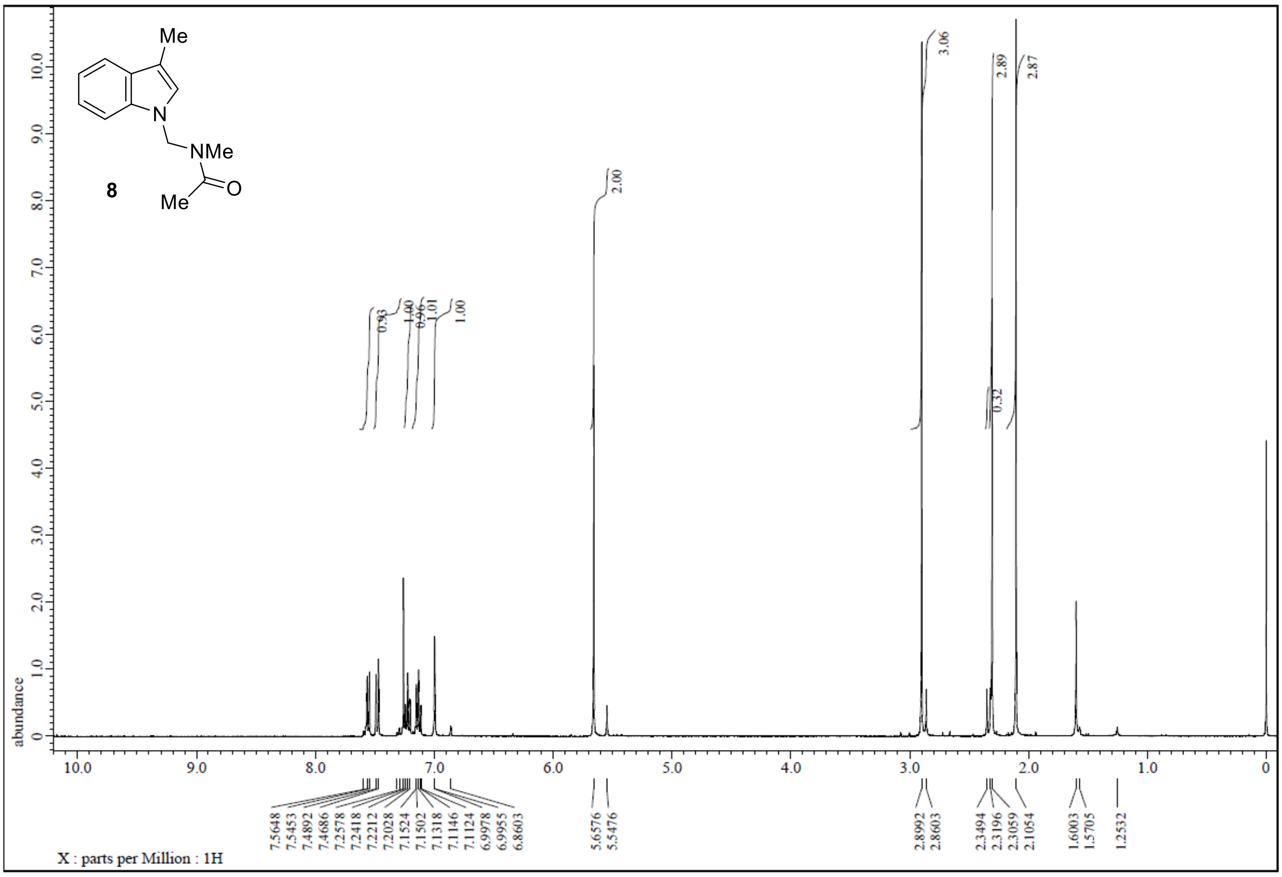

Fig. 9. ¹H NMR (400 MHz; $CDCl_3$) spectrum of *N*-methyl-*N*-[(3-methyl-1*H*-indol-1-yl)methyl]acetamide (**8**).

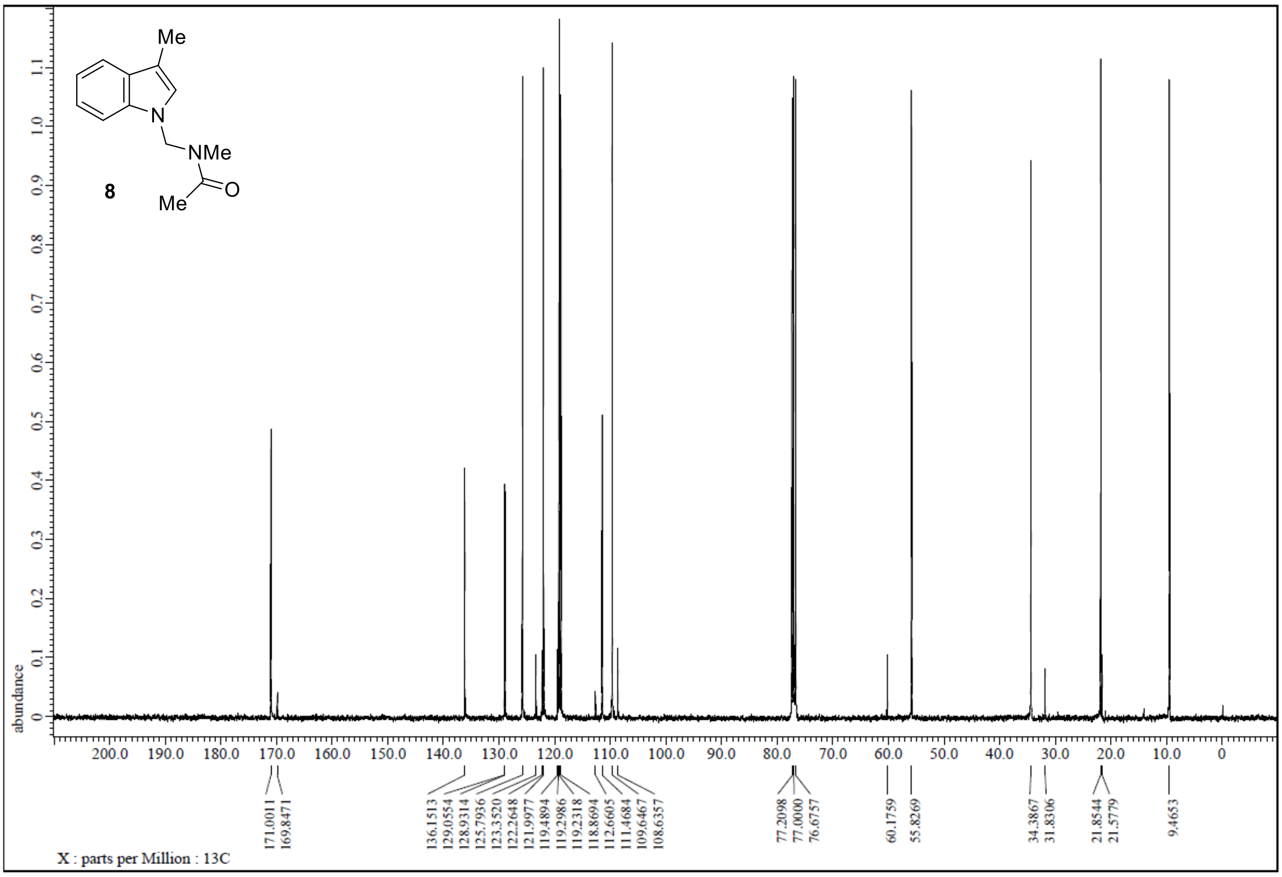

Fig. 10. ^{13}C NMR (100 MHz; $CDCl_3$) spectrum of *N*-methyl-*N*-[(3-methyl-1*H*-indol-1-yl)methyl]acetamide (**8**).
